# The effect of vitamin D and zoledronic acid in bone marrow adiposity in kidney transplant patients: A *post hoc* analysis

**DOI:** 10.1371/journal.pone.0197994

**Published:** 2018-05-25

**Authors:** Mariel J. Hernandez, Luciene M. dos Reis, Igor D. Marques, Maria J. Araujo, Cesar A. M. Truyts, Ivone B. Oliveira, Fellype C. Barreto, Elias David-Neto, Melani R. Custodio, Rosa M. Moyses, Ezequiel Bellorin-Font, Vanda Jorgetti

**Affiliations:** 1 LIM 16 – Laboratorio de Fisiopatologia Renal, Hospital das Clinicas HCFMUSP, Faculdade de Medicina, Universidade de Sao Paulo, Sao Paulo, Sao Paulo, Brasil; 2 Servicio de Nefrología y Trasplante Renal, Hospital Universitario de Caracas, Universidad Central de Venezuela, Caracas, Venezuela; 3 Divisao de Urologia, Hospital das Clinicas, HCFMUSP, Faculdade de Medicina, Universidade de Sao Paulo, Sao Paulo, Sao Paulo, Brasil; 4 Divisao de Nefrologia, Universidade Federal do Parana, Curitiba, Parana, Brasil; 5 Programa de Pos-Graduaçao em Medicina, Universidade Nove de Julho (UNINOVE), Sao Paulo, Sao Paulo, Brasil; 6 Hospital Samaritano Americas Serviços Medicos, Sao Paulo, Sao Paulo, Brasil; Medizinische Universitat Graz, AUSTRIA

## Abstract

**Purpose:**

Osteoblasts and adipocytes are derived from mesenchymal stem cells. An imbalance in the differentiation of these lineages could affect the preservation of bone integrity. Several studies have suggested the importance of this imbalance in the pathogenesis of osteoporosis after kidney transplant (KT), but the role of bone marrow adiposity in this process is not well known, and if the treatment with the anti-absorptive (zoledronic acid-ZA) drugs could attenuate bone loss. Thus, our objective was compare bone marrow adiposity, osteoblasts and osteocytes before and after KT, verify an association between bone remodeling process (Turnover, Volume, and Mineralization—TMV classification), the osteocyte sclerostin expression to evaluate if there is a role of Wnt pathway, as well as the effect of ZA on these cells.

**Methods:**

We studied 29 new living-donor KT patients. One group received ZA at the time of KT plus cholecalciferol for twelve months, and the other group received only cholecalciferol. Bone biopsies were performed at baseline and after 12 months of treatment. Histomorphometric evaluation was performed in bone and bone marrow adipocytes. Sclerostin (Scl) expression in osteocytes was evaluated by immunohistochemistry.

**Results:**

Some bone marrow adiposity parameters were increased before KT. After KT, some of them remained increased and they worsened with the use of ZA. In the baseline, lower bone Volume and Turnover, were associated with increased bone marrow adiposity parameters (some of them). After KT, both groups showed the same associations. Osteocyte Scl expression after KT decreased with the use of ZA. We observed also an inverse association between bone adiposity parameters and lower osteocyte sclerostin expression 12 months after KT.

**Conclusion:**

In conclusion, the present study suggests that KT fails to normalize bone marrow adiposity, and it even gets worse with the use of ZA. Moreover, bone marrow adiposity is inversely associated with bone Volume and Turnover, which seems to be accentuated by the antiresorptive therapy.

## Introduction

Patients with advanced chronic kidney disease (CKD) can be treated with dialysis or renal transplantation. Transplantation is preferable to dialysis, since it corrects most of the symptoms resulting from uremia and leads to greater survival regardless of the patient’s age group [1].

Mineral metabolism disorders, present since the early stages of CKD, lead to biochemical alterations of calcium, phosphorus, parathormone, FGF-23 and vitamin D, in addition to abnormalities in remodeling, mineralization, volume and bone resistance, as well as development of vascular calcifications [2,3]. These disorders are responsible for numerous complications, among them fractures, and increased morbidity and mortality of the patients affected.

Renal transplantation may completely or partially correct biochemical changes, decreasing P and PTH levels, and raising levels of Ca and vitamin D. In the first six months after transplantation, there is a rapid loss of bone mineral density that can continue over the years, leading to osteoporosis, and increased risk of fractures that may be higher than that of dialysis patients. This loss is due to the use of immunosuppressants, especially corticosteroids [4,5].

Rojas et al. have shown a decrease in the number and metabolic activity of osteoblastic cells and an increase in osteoblast apoptosis after KT. In addition, they demonstrated a negative correlation between the osteoblast surface and cumulative doses of glucocorticoids [6]. These and other data suggest that osteoblast dysfunction plays an important role in the pathogenesis of osteoporosis after KT, mainly when pre-existing bone disease and skeletal side effects of immunosuppressive therapy are present [7–9].

Osteocytic proteins expression are altered in CKD patients, amongst them sclerostin [2] an inhibitor of Wnt signaling. Pereira RC, *et al* suggests that the increase in osteocyte sclerostin expression in KT patients may be related to the use of immune-suppressive medications probably contributing to bone loss observed in these patients [10]. Sclerostin is traditionally characterized as inhibitor of osteoblastogenesis (Wnt pathway) is being investigated for new roles in adipocytes development and bone marrow maintenance [11].

In the bone marrow, osteoblasts and adipocytes are derived from the same precursor cell type, mesenchymal stem cells (MSCs) [12]. Adipocyte is an abundant cell in bone marrow and an increased adipose tissue in this compartment has been associated with low bone mass. Several studies suggest that direct differentiation toward the osteoblastic or adipocytic lineage is critical for the structural integrity of the bone and that this process is affected by factors such as aging, menopause, anorexia nervosa, glucocorticoids, and thiazolidinediones, among others [13,14]. Cohen et al. demonstrated an inverse association between these cell lineages by analyzing bone biopsies from osteoporotic women compared with controls [13].

The pathophysiology of post-transplant bone disease is complex and multifactorial most of the time resulting in bone loss. We hypothesized that marrow adiposity would be higher in KT patients due to an imbalance in the process of MSC differentiation favoring adipogenesis contributing to bone loss in these patients and that sclerostin would be involved.

Our group conducted a prospective study in which we evaluated newly transplanted patients who were randomized into two groups. One received bisphosphonate (zoledronic acid) and vitamin D and the other received vitamin D only. The immunosuppressive scheme was similar and patients underwent bone biopsy in the beginning and one year after kidney transplantation (KT). Thus, the objective of this study was to quantify and verify if there was a difference in bone marrow adiposity parameters (number, perimeter and area), osteoblasts and osteocytes before and after KT in bone biopsies, to evaluate whether there was an association between them with bone Turnover, Mineralization and Volume, to assess osteocyte sclerostin expression, and to evaluate the effect of zoledronic acid on the same parameters.

## Materials and methods

### Subjects and study design

**Fig 1:**
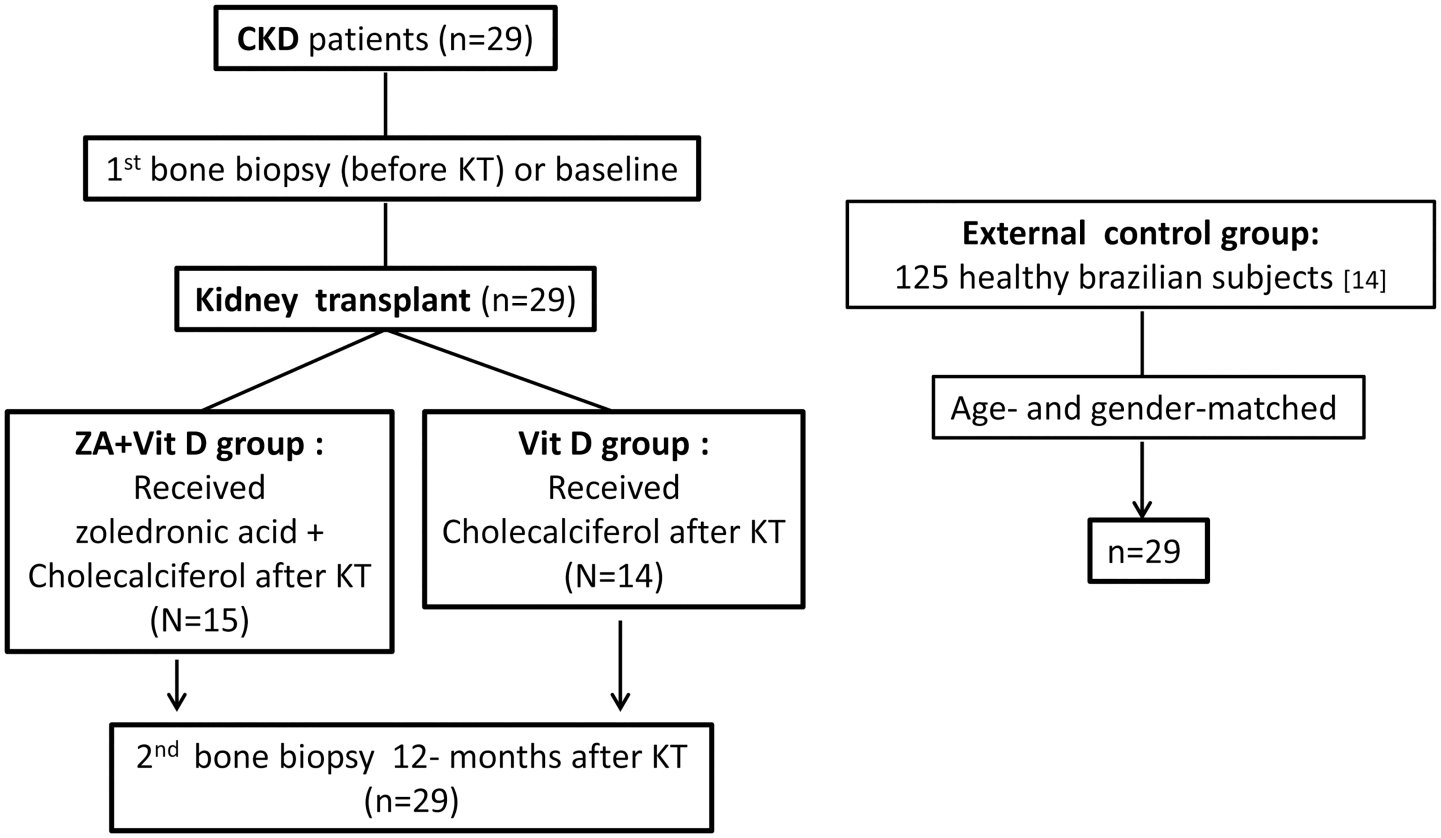
Patients and external controls disposition. CKD: chronic kidney disease, KT: kidney transplant, ZA: zoledronic acid.

This is a *post hoc* analysis of an open-label, prospective and randomized trial (https://clinicaltrials.gov/show/NCT01675089) evaluating the effects of zoledronic acid (ZA) and cholecalciferol compared with cholecalciferol alone to prevent bone loss during the first year after KT. Part of the baseline of this trial was published [15] and the original article was submitted. We studied 29 CKD patients aged 42.1 ± 11.6 (24–61) years, who received a KT from a living donor, with GFR >60 ml/min/1.73m^2^ one year after KT. One group (ZA + Vit D; n = 15) received intravenous ZA (5 mg) in a single dose at the time of KT plus cholecalciferol (25,000 UI) every 15 days for 12 months, and the other group (Vit D, n = 14 patients) received only cholecalciferol (25,000 UI) every 15 days for 12 months. Bone biopsies were performed at the moment of transplantation (baseline) and after 12 months. The bone marrow adiposity evaluated in bone biopsies of the patients (baseline and after KT) was also compared with that of an external control group comprising 29 age- and gender-matched iliac crest bone samples obtained from healthy individuals (Fig 1) [16].

Prednisone was used as a maintenance immunosuppressive therapy in 96.6% of the patients, along with mycophenolic acid and tacrolimus.

Informed consent was obtained from all participants included in the study. The study complied with the Declaration of Helsinki. The Hospital das Clinicas Ethics Committee in Research approved the study (CAPPesq number: 0776/11).

### Serum biochemistry

Blood samples were obtained in a fasting state before bone biopsy. Serum levels of calcium (Ca, reference range: RR: 8.6–10.2 mg/dL), phosphorus (P, RR: 2.7–4.5 mg/dL), and total alkaline phosphatase (ALP, RR: 35–130 UI/L) were determined using routine laboratory methods. Intact parathyroid hormone levels (iPTH, RR: 10–65 pg/mL) were measured using a chemiluminescence assay (DPC, Medlab, San Antonio, USA). Serum 25-hydroxyvitamin D3 (25(OH)D, sufficiency > 30 ng/mL) was measured using a radioimmunoassay (DiaSorin, Stillwater, MN, USA). Bone alkaline phosphatase (BAP, RR: 11.6–42.7 UI/L) and tartrate-resistant acid phosphatase isoform 5b (TRAP5b, RR: 1.5–5.8 UI/L) were measured using an enzyme immunoassay (Metra Biosystems, Mountain View, USA). Serum sclerostin (Scl, RR: 0.42–0.80 ng/mL) was determined using an enzyme immunoassay (Quidel Corporation-TECO medical Group, San Diego, USA). Intra- and inter- coefficients of variability is <5%.

### Bone biopsy and histomorphometric analysis

Bone biopsies were obtained according to a previously described technique [8]. In brief, a transiliac bone biopsy was performed using a Bordier trephine, following a course of doublelabeling tetracycline (20 mg/kg/day) for 3 days, with a 10-day interval. The biopsy was performed 3–5 days after the last dose of tetracycline. Undecalcified specimens were fixed in 70% ethanol, dehydrated, and embedded in methyl methacrylate; 5-μm-thick sections were cut using a Polycut S equipped with a tungsten carbide knife (Leica, Heidelberg, Germany), and some sections were stained with 0.1% toluidine blue, pH 6.4, for the analysis of static and bone marrow parameters. Unstained 10-μm-thick sections were obtained for the analysis of dynamic parameters by microscopy with ultraviolet light. All histomorphometric analysis were performed using a semi-automatic image analyzer and OsteoMeasure software (OsteoMetrics Inc., Atlanta, USA), at 125x magnification.

The following histomorphometric parameters were measured: bone formation rate per unit of bone surface (BFR/BS, μm^3^/μm^2^/day)–defined as the amount of the cancellous bone surface that are replaced per day—This is calculated as the product of the cancellous bone surface covered by the double labels and half of the single labels (MS/BS—mineralizing surface), and by the distance between labels at the doubly labeled surfaces divided by its interval in days (MAR—mineral apposition rate); mineralization lag time (Mlt, days)–defined as the mean time interval, in days, between osteoid deposition and mineralization. This is calculated as O.Th—osteoid thickness by the product of MAR and MS/OS—mineralizing surface related to osteoid surface [17]; bone volume (BV/TV, %)—percentage of the bone area measured per tissue area analyzed; osteoblast number (N.Ob/BS, n°/mm)—total osteoblast number measured per bone perimeter; and osteocyte number (N.Ot/B.Ar, n°/mm^2^)—total osteocyte number measured per square millimeter of the bone area. These parameters followed the guidelines of the American Society for Bone and Mineral Research [18]. The normal values used for static parameters were obtained from our normal laboratory controls [16], whereas the normal range for the dynamic parameters was the same as that described elsewhere [19,20].

The bone histology was categorized according to the newly proposed Turnover/Mineralization/Volume (TMV) system [21]. TMV classification was based on the following parameters: bone turnover (T), defined by BFR/BS (normal values for males: 0.13 ± 0.07; for females: 0.07 ± 0.03) [19]; bone mineralization (M), defined by Mlt (normal values for males and females 23.5±16) [20]; and bone volume (V), defined by BV/TV (normal values for males: 24.0 ± 6.1%; for females: 21.8 ± 7.2) [16]. High turnover defined as BFR/BS values > 1 SD above the normal range, and low turnover as BFR/BS values >1 SD below the normal range. Abnormal mineralization defined as Mlt ≥ 50 days. High bone volume defined as BV/TV values > 1 SD above the normal range, and low bone volume when BV/TV > 1 SD below the normal range.

The bone marrow adiposity analysis was performed at 125x magnification including the whole bone biopsy section stained with 0.1% toluidine blue (Sigma-Aldrich, Saint Louis, USA), at pH 6.4. Complete sections of the bone compartment, comprising the marrow and cancellous bone were analyzed using the OsteoMeasure analysis system (OsteoMetrics Inc., Atlanta, USA). Adipocytes were identified according to the criteria used by Menagh et al. [22]; these cells were defined morphologically as large circular or oval shapes bordered by a prominent cell membrane with the absence of cytoplasmic staining due to the embedding procedure. The following adiposity parameters were analyzed: Ad.Ar/T.Ar (%)–the percentage of the total adipocyte area measured per tissue area analyzed; N.Ad/T.Ar (n°/mm^2^)–total adipocyte number measured per square millimeter of the analyzed tissue; Ad.V/Ma.V (%)–the percentage of the adipocyte volume per marrow volume; and Ad.D (n°/mm^2^)–the adipocyte density, i.e., the adipocyte number per square millimeter of marrow tissue area in the analyzed fields [13].

### Immunohistochemistry

The bone immunohistochemistry (IHC) was performed as previously described [2,23,24]. In brief, 5-μm sections of bone tissue were deplasticized in xylene and chloroform, rehydrated in graded alcohol solutions and partially decalcified in 1% acetic acid. Endogenous peroxidase activity was inhibited by a mixture of 3% hydrogen peroxide in methanol for 30 min, followed by two water washes. Non-specific binding was blocked with an avidin-biotin solution and with 5% normal horse serum with 1% bovine serum albumin. The sections were incubated with affinity-purified monoclonal mouse anti-human sclerostin (Scl) (primary) antibody (R&D Systems, Minneapolis, USA) (dilution 1:100) overnight at 4°C in a humidified chamber. They were then incubated with biotinylated anti-mouse antibody (Vector Laboratories Inc., Burlingame, USA) (dilution 1:200). Next, the sections were incubated for 45 minutes with ABC Complex/HRP–Avidin/Biotinylated enzyme complex kit (Vector Laboratories) followed by AEC (3-Amino-9-ethylcarbazole) chromogenic substrate (Sigma-Aldrich, Saint Louis, USA), and were then counterstained with Mayer’s hematoxylin (Merck KGaA, Darmstadt, Germany). Negative controls were prepared for each bone section by omitting the primary antibody. To quantify the bone expression of sclerostin in osteocytes, staining was classified as either “present” or “absent” in any given osteocyte in trabecular bone [2]. The total osteocyte number per bone area analyzed (N.Ot/B.Ar, n°/mm^2^) was determined at 125x magnification using a semi-automatic image analyzer and OsteoMeasure software (OsteoMetrics Inc., Atlanta, USA). Then, the percentage of osteocytes positive for sclerostin (Scl) relative to the total osteocyte number was calculated (% Scl—percentage of sclerostin expression in osteocyte).

### Statistical analysis

Data are expressed as mean and standard deviations or frequencies and percentages, where appropriate. Linear mixed-effect model (LMEM) was used to compare variables group means: Baseline vs. 12 months after KTx, ZA+Vit D vs. Vit D, Low vs. Normal/High Volume, or Low vs. Normal/High Turnover, with the appropriate contrasts. Each measurement was included as a continuous outcome, with a fixed effect for the corresponding gender or age, and subjects were treated as random effects. Chi-square tests were applied to compare proportions between groups. Pearson’s correlation was used to test the correlation between osteocyte Scl expression and bone marrow adiposity parameters.

Basic statistical data, chi-square test and Pearson’s correlation test were calculated with the SPSS 13.0 software (SPSS Inc., Chicago, IL, USA). Linear mixed-effect models were calculated with the lmer function available in the lme4 and lmerTest packages for R software (R Foundation for Statistical Computing, Vienna, Austria). All statistical tests were two-tailed. A 5% significance level is used for all comparisons.

## Results

### Clinical, biochemical and TMV characteristics

The overall patient mean age was 42 ± 11 years; 62% were male and 93.1% were undergoing hemodialysis therapy, while only 1 patient was undergoing conservative therapy, and 1 was under peritoneal dialysis therapy before the transplantation. The causes of renal failure included hypertension (3; 10%), diabetes mellitus (2; 7%), glomerulonephritis (8; 27%), polycystic kidney disease (1; 4%), urological disease (2; 7%), and unknown (13; 45%).

The biochemical data and bone histomorphometry results are shown in the Table 1. In the Vit D group, only serum Ca increased after KT, however, in ZA+Vit D group, Ca and 25(OH)D increased, P, iPTH, and TRAP5b decreased. The comparison between Vit D and ZA+Vit D groups showed an increase of Ca, P, iPTH, and 25(OH)D and a decrease of TRAP5b. We also found an association between 25(OH)D and N.Ob/BS (r = 0.52; p = 0.02), in Vit D group.

**Table 1:** Biochemical data and bone histomorphometry at baseline and 12 months after kidney transplantation.

	Vit D (N = 14)		p value	ZA + Vit D (N = 15)		p value
	Baseline	12 mo after KT		Baseline	12 mo after KT	
**Laboratory parameters**						
Ca (mg/dL)	8.35 ± 0.56	9.50 ± 0.47	0.001	8.46 ± 0.59	9.53 ± 0.65*	0.0001
P (mg/dL)	3.43 ± 1.84	3.07 ± 0.70	0.1632	3.38 ± 1.42	3.14 ± 0.81*	0.0006
ALP (UI/L)	155.87 ± 42.40	115 ± 60.91	0.2568	97.36 ± 42.91	70.92 ± 22.06	0.0553
iPTH (pg/mL)	538.37 ± 436.59	70.85 ± 34.12	0.599	381 ± 270.19	90 ± 61.63*	0.0018
25(OH)D (ng/mL)	27.45 ± 7.40	29.85 ± 14.32	0.0932	23.20 ± 15.71	30.07 ± 7.95*	0.0074
Scl (ng/mL)	1.09 ± 0.62	0.49 ± 0.15	0.265	1.58 ± 1.14	0.54 ± 0.15	0.513
BAP (UI/L)	138.67 ± 62.04	56.64 ± 41.16	0.168	95.69 ± 62.92	22.78 ± 6.63	0.0785
TRAP5b (UI/L)	12.39 ± 4.21	4.82 ± 2.64	0.149	8.45 ± 3.44	2.34 ± 1.50*	0.007
**Histomorphometry analysis**						
Bone volume (BV/TV) %	27.29 ± 7.56	25.86 ± 8.89	0.001	22.48 ± 4.58	19.91 ± 5.75*	0.0001
Osteoid thickness (O.Th) μm	13.10 ± 3.59	10.26 ± 4.94	0.1552	9.16 ± 3.54	7.88 ± 3.31*	0.0014
Mineralizing surface (MS/BS) %	8.71 ± 3.14	4.09 ± 1.27	0.388	6.21 ± 2.97	3.65 ± 1.36	0.0344
Mineral apposition rate (MAR) μm/day	1.20 ± 0.25	1.14 ± 0.41	0.0031	1.13 ± 0.44	1.19 ± 0.58*	0.0581
Bone formation rate (BFR/BS) μm^3^/ μm^2^/day	0.06 ± 0.06	0.03 ± 0.03	0.3876	0.07 ± 0.08	0.04 ± 0.05	0.155
Mineralization lag time (Mlt) days	63.55 ± 39.75	107.99 ± 159.22	0.1842	73.66 ± 60.42	30.03 ± 18.51 *	0.0895

Data are expressed as the mean ± SD, N (percentage).

Abbreviations: Ca: calcium; P: phosphorus; ALP: alkaline phosphatase: iPTH: intact parathormone; Scl: serum sclerostin; BAP: bone alkaline phosphatase; 25(OH)D: 25hydroxyvitamin D3; TRAP5b: tartrate-resistant alkaline phosphatase; Vit D: cholecalciferol; ZA+Vit D: zoledronic acid + colecalciferol

*p<0.01 ZA+Vit D vs. Vit D.

Regarding bone histomorphometry, in the Vit D group there were a decrease of BV/TV and MAR and in the ZA+ Vit D group, BV/TV, O.Th and MS/BS decreased after KT. The comparison between both groups showed a decrease of BV/TV, O.Th, MAR and Mlt one year after KT.

**Fig 2:**
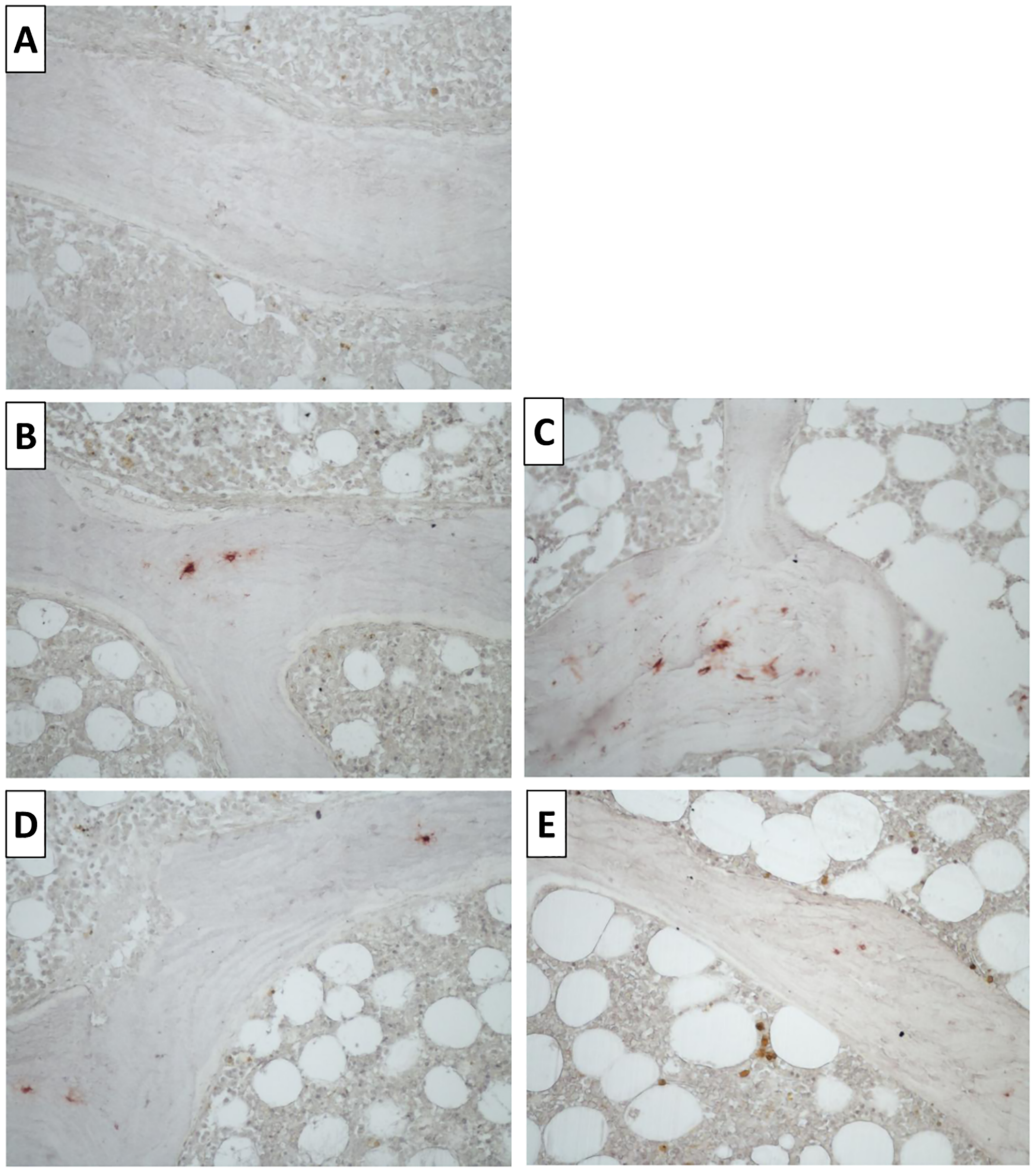
Representative microscopic features of osteocytes SOST expression by immunohistochemistry. In (A) negative control, (B) baseline of Vit D patient, (C) Vit D patient after KT, (D) baseline of ZA+Vit D patient, and (E) ZA+Vit D patient after KT (200x magnification).

The results of TMV classification and immunohistochemistry are shown in Table 2. After KT, Bone Turnover (T) worsened in ZA+Vit D, and the comparison between both groups showed that there was a delayed Mineralization (M) in the ZA+Vit D group. Osteocyte sclerostin expression was decreased in the ZA+Vit group after KT when we compared with Vit D (Fig 2).

**Table 2:** Bone TMV classification and immunohistochemistry at baseline and 12 months after kidney transplantation.

	Vit D (N = 14)			ZA + Vit D (N = 15)		
	Baseline	12 mo after KT	p value	Baseline	12 mo after KT	p value
**Bone biopsy**						
*Volume*						
Low	1 (7.1%)	2 (14.3%)	0.450	1 (6.7%)	4 (26.7%)	0.142
Normal or High	13 (92.9%)	12 (85.7%)		14 (93.3%)	11 (73.3%)	
*Turnover*						
Low	6 (42.9%)	8 (57.1%)	0.663	4 (26.6%)	10 (66.7%)	0.028
Normal or High	8 (57.1%)	6 (42.9%)		11 (73.3%)	5 (33.33%)	
*Mineralization*						
Delayed	3 (21.42%)	4 (28.6%)	0.139	6 (40%)	10 (66.7%)*	0.143
Normal	11 (78.6%)	10 (71.42%)		9 (60%)	5 (33.33%)*	
**Bone immunohistochemistry**						
% Scl	15.13 ± 19.47	37.90 ± 29.42	0.0754	7.62 ± 11.43	19.26 ± 15.36*	0.499

Data are expressed as the mean ± SD, N (percentage).

*p<0.05 ZA+Vit D vs. Vit D.

### Bone marrow adiposity parameters

Table 3 shows the bone marrow adiposity in the external controls and the study population at baseline. The results showed a higher Ad.Ar/T.Ar and Ad.V/Ma.V when we compared the external controls and the study population.

**Table 3:** Bone marrow adiposity parameters and N.Ob/BS in external controls and in the study population at baseline.

	External controls (n = 29)	Study population (n = 29)	p value
Ad.Ar/T.Ar (%)	20.09 ± 9.67	24.00 ± 11.9	0.0009
N.Ad/T.Ar (n°/mm^2^)	75.07 ± 27.94	105.96 ± 41.18	0.0616
Ad.V/Ma.V (%)	26.60 ± 11.00	31.45 ± 14.10	0.0002
Ad.D (n°/mm^2^)	100.80 ± 33.76	141.84 ± 57.04	0.0574
N.Ob/BS (n°/mm)	0.37 ± 0.64	6.29 ± 5.77	0.479

Data are expressed as the mean ± SD. Abbreviations: Ad.Ar/T.Ar: adipocyte area/tissue area; N.Ad/T.Ar: adipocyte number/tissue area; Ad.V/Ma.V: adipocyte volume/marrow volume; Ad.D: adipocyte density; N.Ob/BS osteoblast number/bone surface.

The bone marrow adiposity parameters remained higher in both groups of patients, one year after KT, when compared with the external controls, in spite of the correction of the uremic microenvironment in transplanted patients, such as:

1. Ad.Ar/T.Ar (%): Vit D (25.08 ± 6.37) and ZA+Vit D (33.93 ± 12.68) vs. 20.09 ± 9.67, p = 0.08 and p = 0.0001, respectively;
2. N.Ad/T.Ar (n°/mm^2^): Vit D (95.75 ± 27.46) and ZA+ Vit D (128.66 ± 35.62) vs. 75.07 ± 27.94, p = 0.02 and p = 0.0001, respectively;
3. Ad.V/Ma.V (%):Vit D (34.34 ± 6.62) and ZA+ Vit D (41.69 ± 13.74) vs. 26.60 ± 11.00, p = 0.02 and p = 0.0001, respectively;
4. Ad.D (n°/mm^2^): Vit D (130.80 ± 30.91) and ZA+ Vit D (156.32 ± 37.12) vs 100.80 ± 33.76, p = 0.008 and p = 0.0001, respectively.

The results of the bone marrow adiposity parameters in each treatment group are shown in Table 4.

**Table 4:** Bone marrow adiposity parameters, N.Ob/BS and N.Ot/B.Ar at baseline and 12 months after kidney transplantation according to each treatment group.

	Vit D (N = 14)			ZA+ Vit D (N = 15)		
	Baseline	12 mo after KT	p value	Baseline	12 mo after KT	p value
Ad.Ar/T.Ar (%)	23.35 ± 11.87	25.08 ± 6.37	0.117	25.99 ± 11.72	33.93 ± 12.68	0.9731
N.Ad/T.Ar (n°/mm^2^)	98.17 ± 42.18	95.75 ± 27.46	0.0181	119.65 ± 42.72	128.66 ± 35.62*	0.150
Ad.V/Ma.V (%)	31.07 ± 13.50	34.34 ± 6.62	0.0131	33.52 ± 13.99	41.69 ± 13.74*	0.7766
Ad.D (n°/mm^2^)	132.82 ± 51.07	130.80 ± 30.91	0.0013	142.02 ± 99.56	156.32 ± 37.12*	0.0617
N.Ob/BS (n°/mm)	6.14 ± 4.90	1.02 ± 1.62	0.529	5.30 ± 5.17	0.78 ± 0.99	0.291
N.Ot/B.Ar (n°/mm^2^)	121.22 ± 55.53	96.16 ± 50.11	0.341	147.20 ± 49.99	115.21 ± 36.29	0.0014

Data are expressed as the mean ± SD. Abbreviations: Ad.Ar/T.Ar: adipocyte area/tissue area; N.Ad/T.Ar: adipocyte number/tissue area; Ad.V/Ma.V: adipocyte volume/marrow volume; Ad.D: adipocyte density; N.Ob/BS osteoblast number/bone surface; N.Ot/B.Ar: osteocytes number/bone area; KT: kidney transplantation; Vit D: cholecalciferol; ZA+ Vit D: zoledronic acid + cholecalciferol.

*p<0.05 Vit D vs. ZA + Vit D.

In the Vit D group, there was an increase in N.Ad/T.Ar, Ad.V/Ma.V, and Ad.D. On the other side, in the ZA+ Vit D group, compared with the baseline data, there was a decrease in N.Ot/B.Ar. However, when compared Vit D and ZA+ Vit D groups after KT, we found an increase in N.Ad/T.Ar, Ad.V/Ma.V, and Ad.D in the ZA+ Vit D respect to Vit D group.

**Fig 3:**
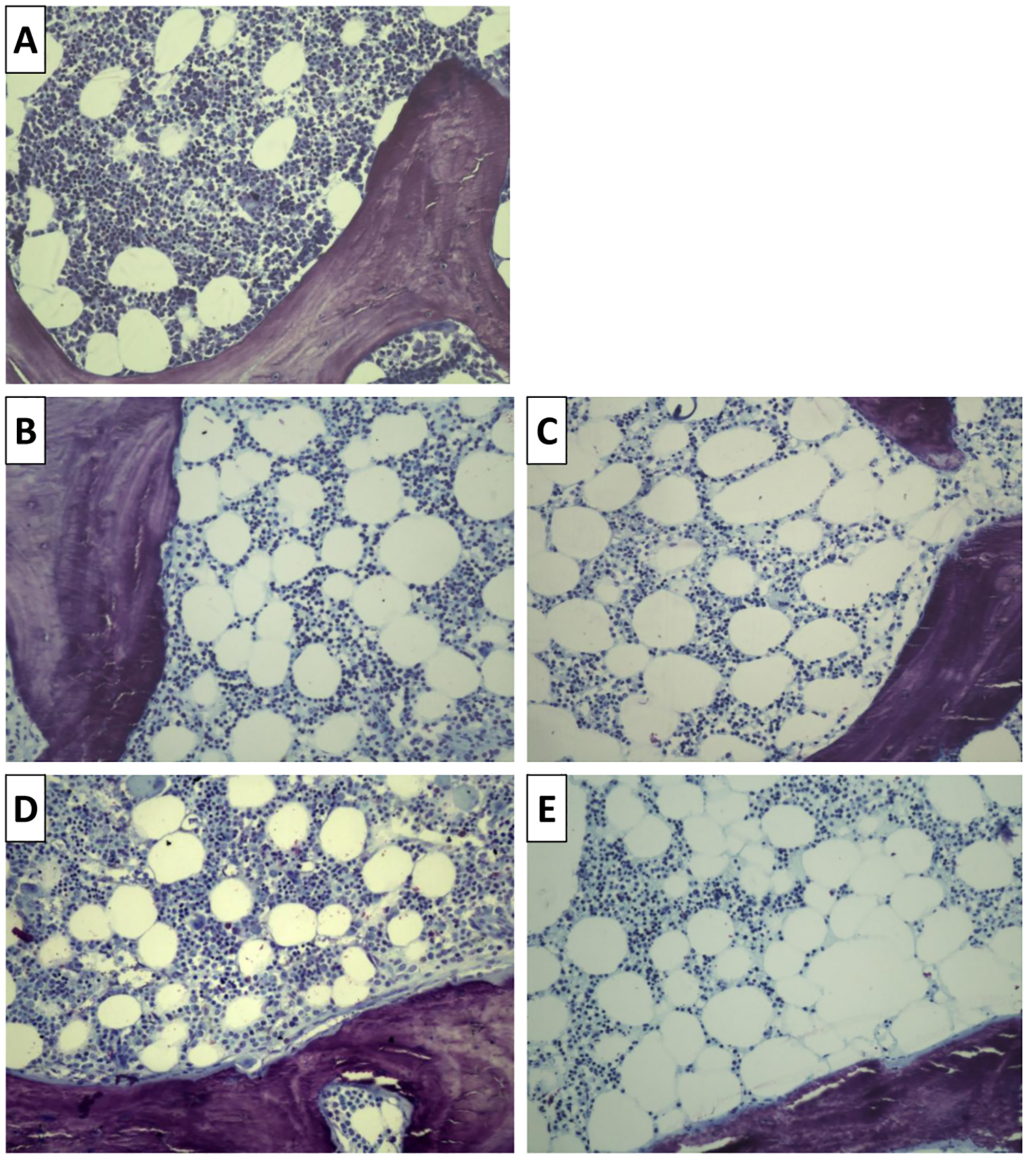
Representative microscopic features of bone marrow. In (A) external control, (B) baseline of Vit D patient, (C) Vit D patient (D) baseline of ZA+ Vit D patient and (E) ZA+ Vit D patient. Toluidine blue staining (200x magnification).

Bone marrow adiposity is shown in Fig 3, and a clear increase in the adipocytic cellularity as well as its distribution regarding the bone trabecular area was evident in both groups of treatment after KT, regarding the baseline and external control group.

### Association between parameters of bone marrow adiposity and bone TMV classification 12 months after KT

As shown in Table 5, we analyzed bone marrow adiposity parameters according to bone TMV classification in both patient groups at 12 months after KT. In the Vit D group, we found that patients with low bone Volume and low bone Turnover presented higher Ad.Ar/T.Ar, Ad.V/Ma.V, and Ad.D values. Concerning the ZA+Vit D group, patients with low bone volume and low turnover showed higher Ad.D.

**Table 5:** Bone marrow adiposity according to bone TMV classification, 12 months after kidney transplantation, by treatment group.

	Vit D group (n = 14)						ZA+ Vit D group (n = 15)					
	Volume		p value	Turnover		p value	Volume		p value	Turnover		p value
	Low (n = 2)	Normal or high (n = 12)		Low (n = 8)	Normal or High (n = 6)		Low (n = 4)	Normal or high (n = 11)		Low (n = 10)	Normal or High (n = 5)	
**Ad.Ar/T.Ar (%)**	34.09 ± 10.21	23.03 ± 8.76	0.0172	25.75 ± 9.91	22.67 ± 8.93	0.0172	36.07 ± 12.87	27.70 ± 12.80	0.6197	35.82 ± 10.38	23.22 ± 12.39	0.8064
**N.Ad/T.Ar (n°/mm**^**2**^**)**	112.63 ± 12.26	92.93 ± 28.59	0.217	94.63 ± 31.42	97.24 ± 23.97	0.0099	135.46 ± 44.49	121.62 ± 35.13	0.104	127.41 ± 38.53	121.12 ± 36.55	0.104
**Ad.V/Ma.V (%)**	38.31 ± 11.05	32.03 ± 10.56	0.0004	33.73 ± 11.34	31.69 ± 10.10	0.0004	41.82 ± 14.19	35.53 ± 14.94	0.5735	44.59 ± 11.05	29.57 ± 14.27	0.6969
**Ad.D (n°/mm**^**2**^**)**	140.54 ± 25.54	130.76 ± 43.20	0.0004	144.72 ± 46.13	138.90 ± 36.47	0.0004	155.60 ± 50.07	150.64 ± 52.28	0.0244	169.56 ± 46.77	135.64 ± 50.79	0.0244
**N.Ob/BS (n°/mm)**	1.22 ± 1.72	0.99 ± 1.68	0.468	0.40 ± 0.56	1.85 ± 2.23	0.432	0.06 ± 0.12	1.00 ± 1.01	0.569	0.77 ± 1.13	0.69 ± 0.56	0.436

Data are expressed as the mean ± SD. Ad.Ar/T.Ar: adipocyte area/tissue area; N.Ad/T.Ar: adipocyte number/tissue area; Ad.V/Ma.V: adipocyte volume/marrow volume; Ad.D: adipocyte density; N.Ob/BS osteoblast number/bone surface.

Overall, at baseline, we found an inverse association between bone volume and Ad.Ar/T.Ar (r = -0.28, p = 0.06). Turnover (BFR/BS) was inversely associated with Ad.Ar/T.Ar (r = -0.44, p = 0.009), Ad.V/Ma.V (r = -0.44, p = 0.009) and Ad.D (r = -0.35, p = 0.03), and directly associated with N.Ob/BS (r = 0.54, p = 0.001).

### Osteocyte Scl expression determined by immunohistochemistry in relation to bone marrow adiposity parameters

When we analyzed all patients at 12 months after KT, we detected an inverse correlation between osteocyte sclerostin expression and bone marrow adiposity parameters (Table 6).

**Table 6:** Correlations between osteocyte Scl expression with bone marrow adiposity and N.Ob/BS, 12 months after KT.

	r	P	95% Confidence Interval
Ad.Ar/T.Ar (%)	-0.33	**0.030**	-0.603; 0.056
N.Ad/T.Ar (n°/mm^2^)	-0.43	**0.009**	-0.713; -0.004
Ad.V/Ma.V (%)	-0.17	0.170	-0.499; 0.248
Ad.D (n°/mm^2^)	-0.37	**0.020**	-0.696; 0.082
N.Ob/BS (n°/mm)	0.04	0.822	-0.427; 0.392

Ad.Ar/T.Ar: adipocyte area/tissue area; N.Ad/T.Ar: adipocyte number/tissue area; Ad.V/Ma.V: adipocyte volume/marrow volume; Ad.D: adipocyte density; and N.Ob/BS: osteoblast number/bone surface.

## Discussion

To our knowledge, this is the first study to analyze histomorphometric changes in bone marrow adiposity in relation to TMV classification in patients before and after KT. Our study showed that, compared with healthy individuals, CKD patients before renal transplantation exhibit a disproportionate increase in bone marrow adiposity, measured as Ad.Ar/T.Ar and Ad.V/Ma.V, predominantly this last finding. A similar finding was recently observed by Moorthi et al. [14], who evaluated bone marrow adiposity in CKD patients using MRS. Another important finding was that KT did not correct these alterations after 12 months, despite of the use of cholecalciferol supplementation associated or not to zoledronic acid. In fact, the addition of ZA resulted in more intense adipogenesis as compared to the Vit D group.

Historically, bone marrow adipose tissue was considered an inert tissue that accumulates in bone marrow to fill the empty space after involution of the hematopoietic tissue, and its association with low bone mass was considered an epiphenomenon. However, an important body of evidence has suggested that adipogenesis is closely related to osteoblastogenesis because they share the same precursor cell type, the MSCs [25]. These are multipotent cells capable of differentiating into osteogenic, chondrogenic, adipogenic, myogenic and neurogenic lineages, depending on the microenvironment and stimuli to which they are exposed [26]. Several factors may determine a disruption of the fine balance between the processes of adipogenic and osteogenic differentiation favoring the former. Some of these factors include aging, estrogen deficiency, diabetes, thiazolidinedione therapy, calorie restriction, as in anorexia nervosa, and the use of some drugs, such as glucocorticoids [13].

Several studies have shown that bone loss begins early after transplantation and may persist for years. The physiopathology of bone loss in KT is complex and multifactorial. Our results provide new insights into the understanding of bone loss and bone marrow adiposity behavior after KT. Our results showed a decrease in BV/TV, in the context of an increase in the adipocyte volume, and it may probably occur via an imbalance in MSC differentiation toward adipogenesis [12,13]. We observed changes in TMV parameters in KT patients, there was an increase in the number of patients with low Turnover in ZA+Vit D group, as well as more patients with delayed Mineralization in those that use ZA, suggesting a synergic effect between the transplant condition and the use of anti-resorptive drugs leading to an over suppression of bone remodeling, leading to bone loss. Moreover, these changes were accompanied by an increase in bone marrow adiposity parameters, which seemed to be more pronounced in the ZA group. In this context, the characteristic distribution of the adipocytes in the peritrabecular palisade may allow us to speculate that its localization in the bone marrow might not be randomly determined, but might reflect the active role of these cells on bone metabolism (Fig 3). In patients with idiopathic osteoporosis, the increase in bone marrow adiposity is associated with bone loss [12,13,27]. Apparently, an increase in adiposity is associated with bone alterations, regardless of the underling cause.

A particular feature of transplantation is the use of immunosuppressive therapy based on glucocorticoids. The prolonged use of these drugs raises another question concerning whether glucocorticoid therapy may be an additional trigger of molecular mechanisms related to osteoblast and osteocyte apoptosis and MSC differentiation toward adipocytes, leading to reduced bone formation and increased osteoclast life-span. These effects may be related to the suppression of the Wnt pathway via higher production of its inhibitors, such as Dickkopf-1 (DKK-1) [28,29,30] and Scl [31]. Bonani et al. [32] reported a decrease of serum sclerostin after KT, likely as a consequence of improved renal function, not observed in our study. The circulating sclerostin concentrations have been reported to vary by sex, age, season and severity of diseases and treatment [33] it may justify the differences in our study and this authors. Despite we did not find significant changes in the bone Scl expression, before and after KT, we observed that the use of ZA reduces its expression when compared with the Vit D group, after KT. As far as we know, bone Scl expression depends, among other factors, on the number of osteocytes, which decrease in the ZA+Vit D group about 20%, leading to attenuate the bone expression of Scl, showing the possible effect of bisphosphonates on osteogenic cellularity. These findings suggesting a role of a compensatory mechanism, possibly in response to osteocyte apoptosis as well as upregulation secondary to lower PTH levels after KT, in the ZA+Vit D group. The trend towards normalization of PTH after KT might be able to lose inhibitory effect on osteocyte cells, probably leading to an increase of bone local levels of sclerostin expression, however, we did not see clearly this finding, because remains attenuated by the use of ZA. In our patients, these findings could express the well-known negative feedback between PTH and sclerostin [34,35]. *In vitro* studies have shown the enhancing effects of glucocorticoids [36] and Scl in bone marrow adipogenesis [31]. Ukita *et al*. [31] demonstrated that Scl positively regulated adipocyte differentiation in 3T3-L1 cells based on the increased expression of adipogenic transcription factors, such as PPARγ and C/EBPβ, during differentiation. In this regard, it was expected an increase of bone Scl expression in our patients and a directly association with bone marrow adiposity parameters; however, our results were the opposite, as an inverse correlation was found in the overall patients after KT. As such, it is necessary to take into account that other pathways may be responsible for MSC differentiation in this context, possibly blocking the Wnt pathway promotes synthesis and adipocyte differentiation. Further studies are needed to clarify these mechanisms.

The decreased osteoblastic activity after transplantation has led to a search for alternative therapeutic strategies. Bisphosphonates have not only an inhibitory effect on osteoclastogenesis but also on bone marrow adipogenesis. Indeed, these drugs blocked adipogenesis and promoted osteoblast differentiation and activity in an *in vitro* model of human MSC differentiation, as measured by the expression of ALP and the mineralization capacity [37,38]. These and other studies give further support to the use of bisphosphonates for the treatment of osteoporosis associated with aging and glucocorticoid, situations in which osteoblasts are replaced by adipocytes [39–43]. Most clinical studies have demonstrated that antiresorptive drugs are capable of slowing the rate of bone loss after transplantation [42,43].

It is noteworthy that in our study, both treatment groups presented bone loss as a decrease in trabecular bone volume, even though the ZA group presented more delayed mineralization and higher bone marrow adiposity than the Vit D group. Contrary to our findings, *in vivo* studies have demonstrated that risedronate and ZA reduces bone marrow adiposity in women with postmenopausal osteoporosis [40] and in ovariectomized rats [38]. Furthermore, previous studies have reported that bisphosphonates ameliorate bone mineralization [44]. Taken together, our findings suggest a possible interference between KT condition and effects of ZA on bone Turnover and Mineralization and MSC differentiation. Actually, a recent study has demonstrated that bone and mineral abnormalities may persist even after two years of a successful KT, including abnormal bone mineralization [8]. Regarding the later, it should be noted that antiresorptive therapy could interfere the osteocyte number and Scl expression, since despite the transplant process remains suppressed with the use of ZA.

Prior studies have shown that 25(OH)D is related to stem cell differentiation, acting as key regulator of adipogenesis [45,46]. Duque et al. demonstrated the potential inhibitory effect of PPARγ on bone in skeletally mature C57BL/6 male mice using 1,25(OH)_2_D_3_ in combination with a bisphenol-A-diglycidyl ether, a known inhibitor of PPARγ, leading to higher osteoblastogenesis and bone formation and to a concomitant decrease on bone marrow adiposity. This finding suggests that the pharmacological inhibition of PPARγ might represent an alternative anabolic therapy for bone loss [47]. These effects have not been demonstrated in humans, though. In our study, 25(OH)D levels were near to the normal range before KT and they improve after cholecalciferol supplementation in the ZA+Vit D group, however, did not seem to be enough to attenuate the bone loss and the gain of bone marrow adiposity. We found a positive correlation between N.Ob/BS and 25(OH)D in the Vit D group (r = 0.52, p = 0.02), suggesting a possible role of this hormone in the process of osteoblastogenesis. Therefore, supplementation with cholecalciferol should be considered as an adjuvant therapy for bone loss in kidney transplant patients.

Our study has certain limitations, such the sample size, *post hoc* design and multifactorial nature of KT. Thus, one should keep in mind that one sole study cannot explain the numerous alterations in bone histology found at 12 months after KT because there are several confounding factors, such as just the CKD and CKD—Mineral and Bone Disorder (CKD-MBD) before transplantation, decreased mobility, nutritional status, initial biochemical parameters and ongoing pharmacotherapy, among others, which can influence bone behavior after transplantation.

In conclusion, the present study suggests that KT fails to normalize bone marrow adiposity, and it even gets worse with the use of strategies that could have ameliorated it, i.e. ZA therapy. Moreover, bone marrow adiposity is inversely associated with bone Volume and Turnover, which seems to be accentuated by the antiresorptive therapy. Unfortunately, our study does not clearly answer the molecular mechanisms involved and further investigation of these mechanisms may help understanding the role of modulators of bone marrow adiposity and may lead to correct the bone abnormalities seen in KT patients.

## Supporting information

S1 Table Study’s data. (XLSX)
